# Immunostimulatory Profile of Cancer Cell Death by the AdV-Lumc007-Derived Oncolytic Virus ‘GoraVir’ in Cultured Pancreatic Cancer Cells

**DOI:** 10.3390/v15020283

**Published:** 2023-01-19

**Authors:** Selas T. F. Bots, Sanne L. Landman, Martijn J. W. E. Rabelink, Diana J. M. van den Wollenberg, Rob C. Hoeben

**Affiliations:** Department of Cell and Chemical Biology, Leiden University Medical Center, 2333 ZC Leiden, The Netherlands

**Keywords:** pancreatic ductal adenocarcinoma, oncolytic virus, non-human primate adenovirus, immunogenic cell death, STING

## Abstract

Pancreatic ductal adenocarcinoma (PDAC) is a highly aggressive malignancy which shows unparalleled therapeutic resistance. Oncolytic viruses have emerged as a new treatment approach and convey their antitumor activity through lysis of cancer cells. The therapeutic efficacy of oncolytic viruses is largely dependent on the induction of immunogenic cell death (ICD) and the subsequent antitumor immune responses. However, the concurrent generation of antiviral immune responses may also limit the a virus’ therapeutic window. GoraVir is a new oncolytic adenovirus derived from the Human Adenovirus B (HAdV-B) isolate AdV-lumc007 which was isolated from a gorilla and has demonstrated excellent lytic activity in both in vitro and in vivo models of PDAC. In this study, we characterized the immunostimulatory profile of cancer cell death induced by GoraVir and the concerted cellular antiviral responses in three conventional pancreatic cancer cell lines. While GoraVir was shown to induce late apoptotic/necrotic cell death at earlier time points post infection than the human adenovirus type 5 (HAdV-C5), similar levels of ICD markers were expressed. Moreover, GoraVir was shown to induce ICD not dependent on STING expression and regardless of subsequent antiviral responses. Together, these data demonstrate that GoraVir is an excellent candidate for use in oncolytic virotherapy.

## 1. Introduction

Pancreatic ductal adenocarcinoma (PDAC) is a highly aggressive malignancy associated with a poor survival rate due to its late detection and therapy resistance. Immunotherapies (e.g., immune checkpoint inhibitors α-PD-1, α-PD-L1, and α-CTL-4) have yielded improvements in overall survival for various advanced and metastatic cancers in selected patient groups [1,2,3]. Unfortunately, immunotherapy as standalone treatments as well as in combination therapies have shown only marginal improvements in survival of patients with PDAC [4]. This has been attributed in part to the immunosuppressive tumor microenvironment shaped by cancer cells, immune cells, as well as cancer-associated fibroblasts (CAFs) [5,6].

Immunogenic cell death (ICD) is a form of cell death which has the capacity to activate adaptive immunity and induce lasting anti-tumor responses [7]. The most commonly described forms of ICD are necroptosis, pyroptosis, and in some cases apoptosis, yet ICD is mostly characterized by the expression of specific danger-associated molecular patterns (DAMPs) [8]. There are several known methods to induce ICD including various chemotherapeutic agents [9], photodynamic therapy [10], physiochemical therapies [11], and oncolytic virotherapy [12].

Oncolytic viruses have emerged as a tool in a new treatment approach, and they convey their antitumor activity through lysis of cancer cells which, upon release of DAMPs and tumor antigens, can initiate an immune response directed against the tumor cells. These viruses have a natural tendency to convert an immunologically suppressed or ‘cold’ tumor to a pro-inflammatory or ‘hot’ state, thereby sensitizing these tumors for subsequent immune checkpoint blockade [13]. GoraVir is a new oncolytic adenovirus derived from the Human Adenovirus B (HAdV-B) isolate AdV-lumc007 which was isolated from a gorilla. GoraVir is adapted to improve its tumor selectivity by removal of the codons for one of the retinoblastoma (Rb)-binding domains in E1A [14]. The virus showed no measurable preexisting neutralizing immunity in humans and demonstrated strong oncolytic potency in multiple tumor types. Moreover, it was shown to kill cancer-associated fibroblasts and delayed tumor growth in a pancreatic cancer xenograft model upon a single-dose administration [15].

The therapeutic efficacy of oncolytic viruses is largely dependent on the induction of ICD and the subsequent antitumor immune responses [16]. However, the induction of antitumor responses inevitably elicits innate and adaptive antiviral immunity. This can lead to viral clearance and loss of therapeutic virus [17]. Meanwhile, cancer cells frequently show defects in the interferon pathway which disrupts their ability to mediate proper antiviral responses [18]. While such defects prolong the window of opportunity for oncolytic viruses to replicate and disseminate, they can influence the virus’ ability to induce ICD [19]. So far, GoraVir has demonstrated excellent lytic activity in vitro yet its ability to induce ICD and its sensitivity to antiviral responses has not been studied. Here, we present the immunostimulatory profile of cancer cell death induced by GoraVir.

## 2. Materials and Methods

### 2.1. Reagents and Buffers

RIPA lysis buffer contains 50 mM Tris·HCl pH 7.5, 150 mM sodium chloride, 0.1% sodium dodecyl sulfate, 0.5% sodium deoxycholate, and 1% NP40. Before use, the buffer was supplemented with protease inhibitors (complete mini tablets, Roche Diagnostics, Almere, The Netherlands) and stored at 4 °C. Western sample buffer has the following ingredients: 50 mM Tris·HCl pH 6.8, 10% glycerol, 2.5% β-mercaptoethanol, 2% SDS, and 0.025% bromophenol blue.

### 2.2. Cell Lines

Human pancreatic cancer cell lines BxPC-3 (CRL-1687), HPAF-II (CRL-1997), and MIA PaCa-2 (CRM-CRL-1420) were all purchased from the ATCC and cultured in high glucose Dulbecco’s modified Eagle’s medium (DMEM, Thermo Fisher Scientific, Bleiswijk, Netherlands) supplemented with 8% fetal bovine serum (FBS, Thermo Fisher Scientific), and 100 IU/mL penicillin and 100 μg/mL streptomycin (P/S). All cells were cultured in an atmosphere of 5% CO_2_ at 37 °C.

### 2.3. Adenoviruses

All experiments were performed with CsCl-purified virus stocks. A detailed outline of the CsCl-purification method for adenoviruses has previously been described [14]. GoraVir is a replication-competent vector derived from the HAdV-B gorilla adenovirus AdV-lumc007 and carries a deletion of the codons for one of the Rb-binding domains of E1A and has been previously described [14]. All infections were performed using DMEM supplemented with 2% FBS and P/S.

### 2.4. Flow Cytometry

Cells were collected via trypsinization and either subjected to the Alexa Fluor 488^®^ annexin V/dead cell apoptosis kit (V13241, Thermo Fisher Scientific, Waltham, MA, USA) according to the manufacturer’s protocol or stained extracellularly with primary antibodies anti-calreticulin (1:50, #PA-900, Invitrogen, Waltham, MA, USA) and anti-HSP70 (1:25, #sc-66048, Santa Cruz, Dallas, TX, USA) according to standardized methods. As a secondary antibody goat-α-mouse PE (1:1000, #12-4010-82, eBioscience, San Diego, CA, USA) and donkey-anti-rabbit Alexa Fluor 488 (1:200, #A-21206, Invitrogen) were used. All samples were analyzed using a LSR-II cytometer (BD) and analyzed using FlowJo software (version 10.8.0).

### 2.5. Quantitative Real-Time PCR (qRT-PCR)

Cells were seeded at 7.5 × 10^5^ cells per well in a 6-well plate in DMEM 8% FBS and incubated overnight (o/n) at an atmosphere of 5% CO_2_ at 37 °C one day prior to infection. Next, cells were infected with virus at MOI 10 in DMEM supplemented with 2% FBS and 1% P/S. At 24 h post infection (hpi), cells were collected via trypsinization and taken up in TRIzol^®^ Reagent (Thermo Fisher Scientific). RNA was extracted by phenol/chloroform extraction and RNA concentrations were measured by NanoDropTM 1000 Spectrophotometer (Thermo Fisher Scientific). For each sample, 1 mg RNA was treated with OPTIZYME DNAse I (Thermo Fisher Scientific) according to manufacturer’s instructions. Next, the RNA was subjected to first-strand cDNA synthesis. The qPCRs were performed using IQ SYBR ^®^ Green Supermix (Bio-Rad, Nazareth, Belgium) and the following primer sets: ISG15-FW 5′-ACCTGACGGTGAAGATGCTG-3′; ISG15-RV 5′-GGTTCGTCGCATTTGTCCAC-3′; ISG54-FW 5′-ATGTGCAACCTACTGGCCTAT-3′; ISG54-RV 5′-TGAGAGTCGGCCCATGTGATA-3′; GAPDH-FW 5′-GCAAATTTCCATGGCACCGT-3′; GAPDH-RV 5′-GCCCCACTTGATTTTGGAGG 3′. A standard qPCR program was followed: 3 min 95 °C; (30 s 95 °C, 15 s 60 °C, 30 s 72 °C × 35 cycles); 10 min 95 °C. Expression of all immunostimulatory genes was normalized to the household gene GAPDH in Bio-Rad CFX Manager 3.1 software. All samples were measured in triplicate.

### 2.6. Adenovirus Genome Copies

Cells were collected via trypsinization and subjected to the mammalian cell lysate protocol of the PurelinkTM genomic DNA kit (K182000, Invitrogen). Genomic DNA was isolated according to the manufacturer’s instructions and DNA concentrations were determined using a NanoDropTM 1000 spectrophotometer (Thermo Fisher Scientific). The concentrations of adenovirus DNA were determined as previously described [14].

### 2.7. Western Blot

Cells were collected via trypsinization and pelleted by centrifugation in a table-top centrifuge at 300× *g* for 5 min. The cell pellets were dissolved in RIPA buffer and incubated at room temperature for 5 min after which insoluble cell debris was removed by centrifugation at 10,000× *g* for 5 min. From the aqueous phase, total protein concentrations were determined using a Bradford protein assay (Bio-Rad, Lunteren, The Netherlands) according to manufacturer’s instructions. Samples were separated by gel electrophoresis using 25 µg protein on a sodium dodecyl sulfate 12% polyacrylamide gel and transferred onto 0.2 µm nitrocellulose membranes using the trans-blot turbo transfer system (Bio-Rad). After blocking, proteins were visualized using standard protocols. Rabbit polyclonal anti-cGAS (#HPAF031700, Atlas Antibodies, Stockholm, Sweden) and rabbit monoclonal anti-STING (#13648, Cell Signaling Technology, Leiden, The Netherlands) antibodies were used at a dilution of 1:1000, and mouse monoclonal anti-Vinculin (#V9131, Sigma-Aldrich, St. Louis, MO, USA) at a dilution of 1:10,000. Proteins were visualized using Clarity™ Western ECL Substrate (Bio-Rad) and images were obtained by using a ChemiDoc MP (Bio-Rad). Protein bands were quantified using Image J software (v1.53a, National Institutes of Health, Bethesda, MD, USA).

### 2.8. Statistical Analyses

All statistical analyses were performed using GraphPad Prism software (v9.0.1, Graphpad Software, La Jolla, CA, USA). Data are presented as mean ± SD unless otherwise stated. Unpaired analyses (one-way ANOVA and unpaired *t* tests) were used for analyses of repeated experiments, and *p* < 0.05 was considered significant throughout. Detailed descriptions about statistical analysis are described in the figure legends. Significant differences are indicated by asterisks, with *p* values < 0.05 shown as *, <0.01 as **, and <0.001 as ***.

## 3. Results

### 3.1. GoraVir Rapidly Induces Late Apoptotic/Necrotic Cell Death

**Figure 1:**
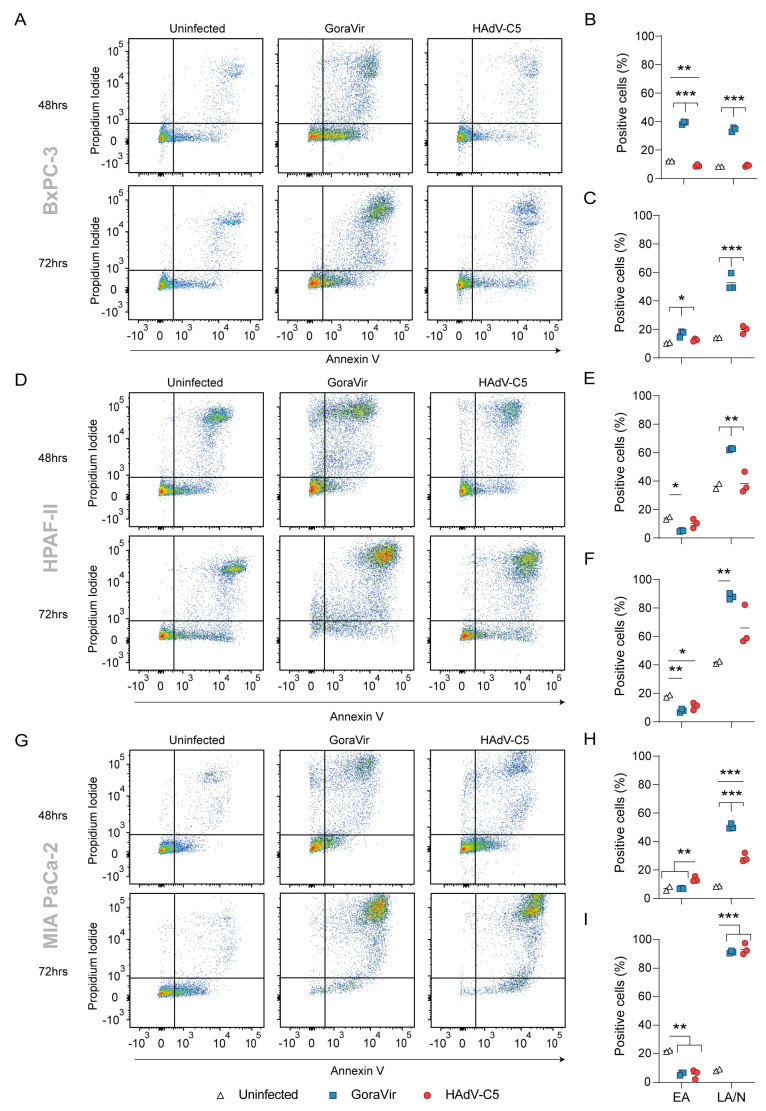
Virus-induced cell death of pancreatic cancer cell lines by GoraVir. Cells were infected with GoraVir or HAdV-C5 at MOI 10 and stained with Annexin V and propidium iodide at 48 and 72 hrs post infection (hpi). Depicted are representative figures out of *n* = 3 for (**A**) BxPC-3, (**D**) HPAF-II, and (**G**) MIA PaCa-2. Annexin V + PI- cells were classified as early apoptotic (EA) cells and Annexin V-PI+ and Annexin V + PI+ together as late apoptotic/necrotic (LA/N) cells. Percentage of single cell population in EA or LA/N was determined at 48 and 72 hpi for BxPC-3 (**B**,**C**), HPAF-II (**E**,**F**), and MIA PaCa-2 (**H**,**I**), respectively. Depicted are means of *n* = 3 and comparisons were performed using one-way ANOVA with Tukey correction for multiple testing. Significant differences are indicated by asterisks, with *p* values < 0.05 shown as *, <0.01 as **, and <0.001 as ***.

Oncolytic viruses are characterized by their capacity to induce a lytic form of cell death, which aids in their subsequent ability to induce adaptive antiviral and anti-tumor immune responses. Conversely, these antiviral responses provide these vectors with a limited therapeutic window [17]. For that reason, an oncolytic vector that progresses to the initiation of cell death quickly after infection could increase the therapeutic efficacy. To characterize the time course of GoraVir-mediated cell killing, three human pancreatic cancer cell lines (BxPC-3, HPAF-II and MIA PaCa-2) were exposed to GoraVir, or as a reference control HAdV-C5, at MOI 10. Virus-induced cell death was monitored at 24, 48, and 72 hpi by flow cytometry using propidium iodide (PI)/Annexin V staining. Annexin V + PI− cells were considered early apoptotic (EA) cells, while Annexin V-PI+ and Annexin V + PI+ cells were considered late apoptotic/necrotic (LA/N) cells. In all cell lines, no induction of cell death was observed for both viruses in comparison to uninfected cells at 24 hpi. At 48 hpi, infection with GoraVir induced significantly higher percentages of LA/N cells than HAdV-C5 in BxPC-3 (Figure 1A,B), HPAF-II (Figure 1D,E), and MIA PaCa-2 cells (Figure 1G,H). For the BxPC-3 cells, these differences were further increased at 72 h, where most cells had shifted to a LA/N type of cell death (52.71 ± 3.43%, Figure 1C). In contrast, HAdV-C5 did not show any increase in EA or LA/N BxPC-3 cells when compared to uninfected cells by 72 hpi.

HPAF-II cells showed an increase in LA/N cells compared to uninfected cells upon infection with GoraVir (88.08 ± 1.24%) as well as HAdV-C5 (65.94 ± 8.20%) at 72 hpi, although the latter was not statistically significant (Figure 1F). It should be noted that uninfected HPAF-II cells already showed a high percentage of LA/N cells (36.38 ± 1.75%). Finally, in MIA PaCa-2 cells, GoraVir and HAdV-C5 induced similar amounts of LA/N cells at 72 hpi (91.35 ± 0.52% and 93.39 ± 2.24%, respectively) (Figure 1I). Taken together, GoraVir was demonstrated to be more potent in inducing LA/N cell death in the BxPC-3 cells and the HPAF-II cell lines than HAdV-C5. While no differences were observed between the two viruses in the MIA PaCa-2 cell line at 72 hpi, a much stronger induction of LA/N cell death was observed earlier, at 48 hpi, upon infection with GoraVir compared to HAdV-C5. Therefore, these data suggest that GoraVir is able to rapidly induce a LA/N phenotype in infected cancer cells and seems to induce cell death faster than HAdV-C5.

### 3.2. Infection with GoraVir Leads to Immunogenic Cell Death

**Figure 2:**
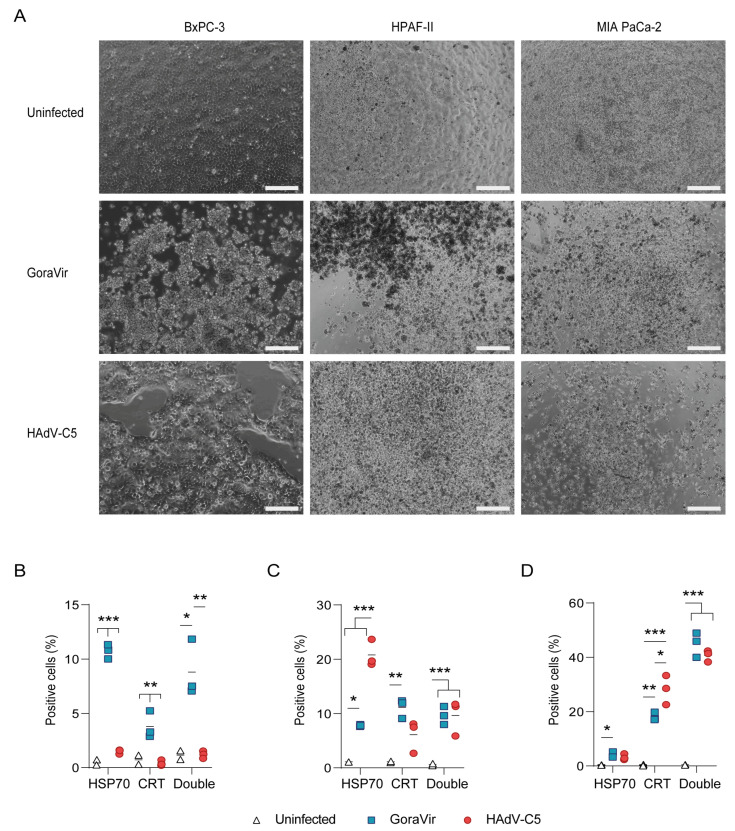
Protein expression of immunogenic cell death (ICD) markers in pancreatic cancer cell lines upon infection with GoraVir and HAdV-C5. (**A**) Light microscopy images of pancreatic cancer cells infected with GoraVir or HAdV-C5 at 72 hpi. Scale bar represents 500 µm; (**B**) BxPC-3, (**C**) HPAF-II, and (**D**) MIA PaCa-2 cells were infected with GoraVir or HAdV-C5 at MOI 10 and 72 hpi. The percentage of calreticulin (CTR)-positive, HSP70-positive, or double-positive cells was determined by flow cytometry. Means are depicted of *n* = 3 biologically independent replicates. Means were compared using one-way ANOVA with Tukey correction for multiple testing. Significant differences are indicated by asterisks, with *p* values < 0.05 shown as *, <0.01 shown as **, and <0.001 shown as ***.

For a dying cell to initiate an adaptive immune response, certain activating or ‘eat-me’ signaling molecules will need to be expressed on the cell surface, e.g., calreticulin (CRT), heat shock protein (HSP) 70/90, or released by the cell, e.g., ATP, HMGB1, and type I interferons (IFNs) [20]. This is referred to as ICD and it is directed at the activation and maturation of dendritic cells, as well as to promote antigen processing and presentation [21]. To determine whether GoraVir’s rapid induction of late-phase cell death was immunogenic, all three cell lines were exposed to GoraVir or HAdV-C5 at MOI 10 and cells were collected at 72 hpi. First, the degree of cytopathic effect (CPE) was assessed. Upon infection with GoraVir, in all cell lines, cells were detached and demonstrated marked CPE at 72 hpi (Figure 2A). For the HPAF-II and MIA PaCa-2 cells, strong CPE was visible upon infection with HAdV-C5. For BxPC-3, no clear CPE was induced upon infection with HAdV-C5 although a clear disruption of the cell layer was visible. These observations correlate with the results in Figure 1. Next, the ectopic expression of ICD markers HSP70 and CRT was measured by flow cytometry. Flow cytometric analyses of the ICD markers showed a significant increase in HSP70 + CRT- (10.72 ± 0.38%), HSP70-CRT+ (3.80 ± 0.72%), as well as HSP70 + CRT+ (8.80 ± 1.52%) in BxPC-3 cells upon infection with GoraVir compared to uninfected cells and HAdV-C5, which did not induce the expression of either HSP70 or CRT in this cell line (Figure 2B). In HPAF-II, both GoraVir and HAdV-C5 induced the expression of HSP70 and CRT (Figure 2C). While the amount of HSP70 + CRT+ was comparable between the two viruses, GoraVir showed a trend towards higher amounts of HSP70-CRT+ cells (11.13 ± 0.47%) while HAdV-C5 induced significantly more HSP70 + CRT- (20.81 ± 1.44%). Infection of MIA PaCa-2 cells with GoraVir and HAdV-C5 yielded high percentages of HSP70 + CRT+ (44.96 ± 2.61% and 40.70 ± 1.21%, respectively), followed by an increase in HSP70-CRT+ cells for both viruses (18.07 ± 0.87% and 28.17 ± 3.10%, respectively and only low amounts of HSP70 + CRT- cells (3.92 ± 0.64% and 3.23 ± 0.64%, respectively) (Figure 2D).

Interestingly, the percentages of positive cells were in some cases lower in the BxPC-3 cells compared to those in HPAF-II-infected and MIA PaCa-2-infected cells. Furthermore, the induction of ICD markers for BxPC-3 and MIA PaCa-2 correlated well with the ability of either virus to induce LA/N cell death at 72 hpi (Figure 1C,I). In the HPAF-II cells however, HAdV-C5 was more capable of inducing HSP70 + CRT- cells upon infection than GoraVir despite the lower amount of LA/N positive cells (Figure 1F). Since GoraVir induces LA/N cell death faster than HAdV-C5, we hypothesized that this might also be reflected in a higher expression of ICD markers at 48 hpi in HPAF-II and MIA PaCa-2 cells. However, no differences were observed at that time point (Supplementary Figure S1). Taken together, it seems that although GoraVir induces rapid LA/N cell death, this is not accompanied by a higher expression of ICD markers HSP70 and CRT as compared to HadV-C5-infected cells. Nevertheless, GoraVir-mediated cell death shows the hallmarks of immunogenic cell death in all three cell lines and is largely comparable to, or in the case of BxPC-3 even superior to, HAdV-C5-mediated cell death.

### 3.3. GoraVir Replicates Efficiently in Pancreatic Cancer Cells

**Figure 3:**
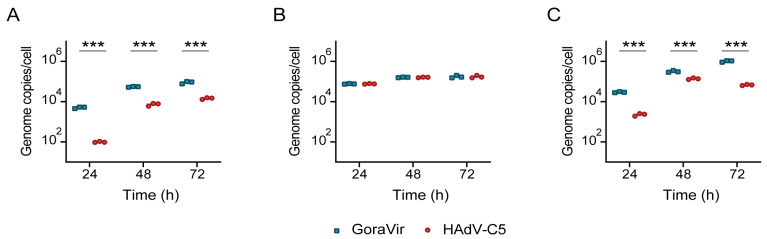
Replication kinetics of GoraVir and HAdV-C5 in pancreatic cancer cell lines. (**A**) BxPC-3, (**B**) HPAF-II, and (**C**) MIA PaCa-2 cells were infected with either GoraVir or HAdV-C5 at MOI 2 and viral genome copies were determined at 24, 48, and 72 hpi. Means are depicted of *n* = 3 biologically independent replicates. Statistical analyses were performed using multiple unpaired *t* tests and the Holm-Šídák correction. Significant differences are indicated by asterisks, with *p* values < 0.001 shown as ***.

The induction of cell death following virus infection has the ability to either limit viral replication or promote viral dissemination. This delicate balance is especially important in the context of oncolytic virotherapy, where these temporal dynamics may influence therapeutic efficacy. In A549 cells, GoraVir has been shown to replicate faster and kill at an earlier onset than HAdV-C5 [14]. Since GoraVir demonstrated earlier onset of cell killing in the pancreatic cancer cell lines, we sought to establish whether this was also accompanied by faster replication kinetics. Therefore, all three cell lines were infected with GoraVir or HAdV-C5 and viral replication was assessed at 24, 48, and 72 h. In BxPC-3 cells, GoraVir replicated significantly better than HAdV-C5 with ~2-log difference in viral genome copies at 24 hpi and ~1-log difference at later time points (Figure 3A). It seems plausible that HAdV-C5′s restrained ability to replicate in these cells underlies its inability to kill these cells within a short time frame (Figure 1C). In HPAF-II cells, infection with both viruses yielded high viral genome copies within the first 24 h of infection which only increased marginally in the following days (Figure 3B). No differences were observed between the viruses. Infection of MIA PaCa-2 cells was comparable to the kinetics observed in BxPC-3 where GoraVir replicates significantly better compared to HAdV-C5 at all time points (Figure 3C). However, the differences between the two viruses were smaller.

Noteworthily, GoraVir was shown to not only replicate faster than HAdV-C5 in two out of three cell lines (i.e., higher viral genome copies at 24 h) but also seemed to generate more viral DNA over the course of infection. This is in line with our previous observations in A549 cells and appears to be a more general characteristic of the virus. While GoraVir’s fast replication kinetics might explain its ability to induce cell death at earlier time points post infection in the BxPC-3 and MIA PaCa-2 cell line (Figure 1B,H), no differences were observed in replication kinetics in the HPAF-II cell line between the two viruses. In conclusion, GoraVir has shown to efficiently replicate in all three pancreatic cell lines with comparable or much faster kinetics than HAdV-C5.

### 3.4. GoraVir Is a Strong Modulator of Antiviral Responses

**Figure 4:**
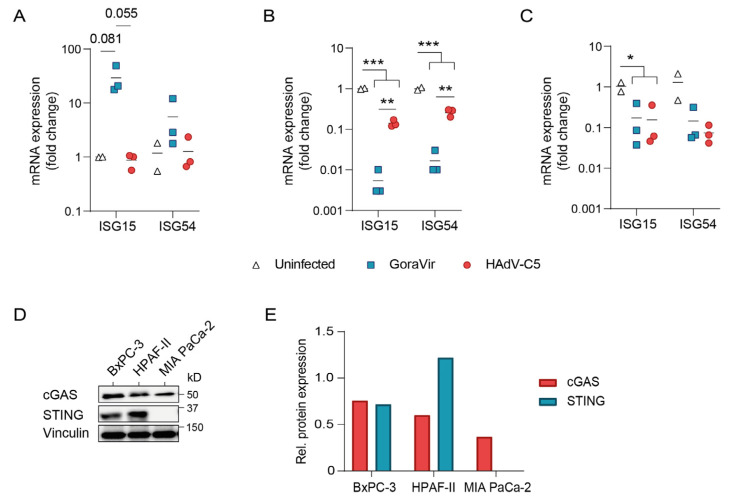
ISG response upon infection with GoraVir or HAdV-C5 through cGAS/STING signaling. (**A**) BxPC-3, (**B**) HPAF-II, and (**C**) MIA PaCa-2 cells were infected with GoraVir or HAdV-C5 at MOI 10 and mRNA expression of ISG15 and ISG54 was measured at 24 h post infection. Fold change expression was calculated relative to GAPDH. Means are depicted of *n* = 3 biologically independent replicates; (**D**) Protein expression of cGAS, STING, and Vinculin in pancreatic cancer cells.; (**E**) Quantification of cGAS and STING protein levels normalized to Vinculin. Means were compared using multiple unpaired t tests and the Holm-Šídák correction. Significant differences are indicated by asterisks, with *p* values < 0.05 shown as *, <0.01 shown as **, and <0.001 shown as ***.

The production of type I IFNs is a key component for the establishment of a pro-inflammatory environment which will allow for the generation of an anti-tumor immune response [22]. However, as induction of an antiviral state following the production of IFNs can hamper viral replication and dissemination, viruses have evolved multiple strategies to inhibit or delay such responses [23]. To determine whether infection with GoraVir induces an antiviral state, IFN-stimulated gene (ISG) mRNA expression was measured at 24 hpi with GoraVir and HAdV-C5, as a reference control. In BxPC-3 cells, infection with GoraVir showed a trend towards upregulation of ISG15 (29.19 ± 10.02-fold change (FC)) as well as ISG54 (5.57 ± 3.26 FC) compared to uninfected cells as well as HAdV-C5-infected cells, nearing statistical significance (*p* = 0.081 and *p* = 0.055, respectively) (Figure 4A). For HAdV-C5, no induction of ISG15 nor ISG54 was observed. Infection of HPAF-II cells with GoraVir resulted in a completely opposite trend, where both ISGs were strongly downregulated by ~100-fold compared to uninfected cells (Figure 4B). Infection with HAdV-C5 also resulted in downregulation of ISG15 and ISG54 (0.14 ± 0.02 FC, *p* < 0.001, and 0.26 ± 0.03 FC, *p* < 0.001, respectively) compared to uninfected cells although not as strong as upon infection with GoraVir. Finally, infection of MIA PaCa-2 with either virus resulted in a minor downregulation of ISG15 and no change in mRNA expression for ISG54 (Figure 4C). In addition, no differences between the viruses were observed.

These distinctive responses may be indicative of different defects in the interferon pathway as is frequently observed in cancer cells [18]. In normal cells, incoming Ad particles are sensed via the cytosolic sensor cGAS which in turn activates the STING pathway, leading to, e.g., the activation of ISGs [24]. Several pancreatic cancer cell lines have previously been shown to lack STING, and while this did not alter their susceptibility to an oncolytic herpesvirus [25] it might underlie the different responses observed here. Therefore, cGAS and STING protein expression was measured in all three cell lines by Western blot analysis. All cell lines expressed cGAS protein but only BxPC-3 and HPAF-II expressed detectable levels of STING (Figure 4D). In addition, cGAS protein levels of MIA PaCa-2 cells were somewhat lower compared to BxPC-3 and HPAF-II cells (Figure 4E). The absence of STING in the MIA PaCa-2 cell line could explain the lack of response to Ad infection (Figure 3C). Likewise, the presence of both cGAS and STING in the BxPC-3 cells allows this cell line to respond to an Ad infection (Figure 3A). Interestingly, HPAF-II cells expressed much higher levels of STING than BxPC-3 cells. However, given the downregulation of ISG mRNA expression upon Ad infection in the HPAF-II cell line it seems plausible that the STING pathway might be defective elsewhere. To our surprise, when correlating the sensitivity of the cell lines to infection, we observed a positive correlation between sensitivity and STING protein levels for GoraVir, but not HAdV-C5 (Supplementary Figure S2). Taken together, it appears that GoraVir induces cell-type specific ISG responses upon infection which seem, at least in part, to be dependent on a functional cGAS/STING pathway. Regardless of the variability between the cell lines, GoraVir induces much stronger, bidirectional ISG responses than HAdV-C5. As such, it appears that GoraVir is the stronger modulator of antiviral responses.

## 4. Discussion

Oncolytic viruses naturally elicit innate and adaptive immune responses which aid in their therapeutic efficacy. However, the concurrent generation of antiviral immune responses may limit the virus´ therapeutic window. In this study, we characterized the immunostimulatory profile of cancer cell death induced by GoraVir, a new gorilla-derived oncolytic adenovirus, and the concerted cellular antiviral responses in three conventional pancreatic cancer cell lines. First, GoraVir was shown to induce LA/N cell death faster in all three cell lines compared to HAdV-C5 (Figure 1B,E,H). In some pancreatic cancer cell lines, this could possibly be attributed to faster replication kinetics of GoraVir (Figure 3A,C). It should be noted that infection efficacy of these viruses can be influenced by the varying receptor expression levels on these cells. While HAdV-C5 makes use of the coxsackie and adenovirus receptor (CAR) for viral entry, we recently established that GoraVir makes use of the complement receptor CD46 [15]. However, while the expression levels of CAR and CD46 differ between the cell lines, their relative expression of these receptors was similar. The observed differences in viral replication and the induction of cell death can be attributed only in part to different expression levels of these receptors. Furthermore, although no differences were observed in replication kinetics in the HPAF-II cell line (Figure 3B), GoraVir induced LA/N cell death more rapidly. Therefore, other factors (e.g., virus release from the cells) will also contribute to GoraVir’s ability to induce LA/N cell death more rapidly than HAdV-C5. For the HAdV-C species, the E3 conserved region (CR) 1β gene encodes the adenovirus death protein (ADP or E3-11.6K), which is highly expressed during the late stages of infection and appears to be essential for lytic infection of cells [26]. The E3-CR1 comprises a species–specific combination of genes (CR1α, β, γ and/or δ), which show little or no homology between HAdV species [27] and may have distinct biological functions [28]. A few attempts have been made to identify a protein of similar function to ADP in the HAdV-B species, but so far they have been unsuccessful [27,29]. While the open reading frames of the E3 gene region are relatively conserved between human Ads and nonhuman primate Ads of the same species, this region showed a higher variability between Ad types derived from human and nonhuman primate origin than human origin alone [14,30]. Gaining more insight into the functional significance of these genes could aid in improving our understanding of oncolytic adenovirus vectors derived from other types than HAdV-C5.

Second, despite the rapid induction of LA/N cell death, no enhanced expression of ICD markers was observed for HPAF-II and MIA PaCa-2 cells upon infection with GoraVir compared to HAdV-C5 (Figure 2). This suggests that the expression of ICD markers is not strongly linked to the stage of cell death. HAdV-C5 has previously been shown to induce cell-dependent ectopic expression of CRT and HSP90 as well as the release of HMBG1 and extracellular ATP in vitro [12,31]. This was shown to induce dendritic cell activation [12]. The observation that GoraVir produces similar levels of ICD as HAdV-C5 is promising for its use as an immunostimulatory agent. Additionally, the absence of STING did not alter the ability of GoraVir and HAdV-C5 to induce ICD. Similarly, the FDA-approved oncolytic herpes simplex virus 1 (HSV) T-VEC was shown to mediate T-cell anti-tumor immunity in a murine melanoma model with low STING expression [32]. On the other hand, the use of R-LM113, another oncolytic HSV retargeted to HER-2, showed reduced immunogenicity due to improper induction of ICD in STING-KO murine cells [19]. Whether these different outcomes are due to the use of STING-deficient as opposed to low-expressing cells, or whether they involve virus or cell-type-specific differences remains to be determined. Of note, STING expression is frequently lost or repressed in cancer cells to avoid senescence upon DNA damage and promotes tumor progression [33]. The STING-independent induction of ICD by Ads might provide the virus with a unique advantage with respect to other oncolytic DNA viruses.

Furthermore, we characterized the strength of the innate antiviral responses elicited upon infection with GoraVir. GoraVir was shown to upregulate or downregulate ISG responses in a cell-dependent manner much more stongly than HAdV-C5 (Figure 4A,C). Type-specific differences in the induction of type I IFN responses have previously been reported for other members of HAdV-B and C species. For example, HAdV-B7 and HAdV-B35 induce much higher gene expression of IFNβ, ISG54, and ISG15 compared to HAdV-C2 at 6 hpi [34]. Since the levels of antiviral responses elicited by the HAdV-B viruses were disproportionally high with respect to viral uptake, the authors related these differences to a (sero)type-specific capsid function involved in virus entry and/or escape. In favor of the latter, GoraVir and HAdV-B7 make use of different receptors for viral entry, i.e., CD46 and desmoglein-2 [35], respectively. The implications of these stronger and, more importantly, cell-dependent bidirectional effects on ISG expression upon infection with GoraVir on the generation of a cellular (anti-tumor) immune response remain to be established.

Unexpectedly, GoraVir replicated with similar efficiency in all three cell lines regardless of the initiated antiviral responses. For C-REV (a mutant oncolytic HSV-1 virus, formerly known as HF10), the induction of virus-mediated cell killing despite antiviral responses was attributed to its ability to suppress STING activation upon infection [25]. Similarly, the HAdV-C5 E1A gene has been demonstrated to antagonize the cGAS/STING pathway by directly binding to STING using a LXCXE motif [36]. This specific domain in E1A is better known for its binding to the retinoblastoma (Rb) protein as to stimulate cell-cycle progression. This region was deleted in GoraVir in an effort to improve its tumor-cell selectivity [14]. However, GoraVir’s E1A contains two additional LXCXE motifs (one more than HAdV-C5), which might have a similar function. It therefore remains possible that the E1A gene of GoraVir modulates STING-mediated signaling.

Activation of the STING pathway plays a critical role in resistance to oncolytic virotherapy [37]. In line with this, T-VEC-mediated cell killing was demonstrated to be inversely correlated to STING expression in melanoma cells [32]. Interestingly, we observed no correlation between STING expression and susceptibility to infection for HAdV-C5 (Supplementary Figure S2B). Likewise, susceptibility to C-REV was not correlated with STING expression [25]. Although many cancer cells lose STING expression entirely, suppression of the pathway via other mechanisms could explain why sometimes no direct correlation can be found [38]. In light of this, it seems striking that susceptibility to infection with GoraVir was positively correlated with STING protein expression and not correlated with the induction of ISGs (Supplementary Figure S2A). The positive correlation with STING expression regardless of the initiated antiviral responses could indicate that STING is involved in GoraVir infection via a different mechanism. Viruses are known to utilize a broad range of cellular proteins to facilitate viral infection, which in some cases are independent from their cellular function [39,40,41]. Interestingly, STING has a non-canonical role in promoting cell death [42]. For example, the magnitude of STING signaling was shown to alter its function and initiate apoptosis in T lymphocytes [43]. Taken together, it seems possible that STING fulfills a role during GoraVir infection distinct from its function in innate immune signaling.

## 5. Conclusions

In conclusion, the ability of GoraVir to efficiently replicate in pancreatic cancer cell lines regardless of subsequent antiviral responses and to induce ICD irrespective of STING expression, seems illustrative of an interesting oncolytic candidate. Since many tumors lose or repress STING, and therefore the ability to generate antiviral responses, GoraVir’s ability to replicate despite these responses might seem futile. However, the tumor microenvironment consists of a myriad of different cell types which can contribute to the establishment of an antiviral landscape. More recently, the production of IFNs by CAFs was shown to limit the efficacy of oncolytic viruses [44]. Contact-dependent cytoplasmic transfer of cGAMP from cancer cells to CAFs was shown to activate the intact STING pathway in these cells, thereby leading to high expression of IFNβ and ISGs. To this end, the versatilely of GoraVir to kill despite the initiation of (STING-mediated) antiviral responses could prolong its therapeutic window and enable the flexible use of combination immunotherapy in pancreatic cancer.

## Data Availability

The datasets generated during and/or analyzed during the current study are available from the corresponding author on reasonable request.

## Supplementary Materials

The following supporting information can be downloaded at https://www.mdpi.com/article/10.3390/v15020283/s1, Figure S1: Protein expression of immunogenic cell death (ICD) markers in HPAF-II and MIA PaCa-2 upon infection with GoraVir and HAdV-C5; Figure S2: Cell viability of PDAC cell lines upon infection with GoraVir and HAdV-C5.
