# Supplementary material for: Immunostimulatory Profile of Cancer Cell Death by the AdV-Lumc007-Derived Oncolytic Virus ‘GoraVir’ in Cultured Pancreatic Cancer Cells
Source: Viruses. 2023 Jan 19;15(2):283. doi: 10.3390/v15020283 (PMC9959036; doi:10.3390/v15020283)
Supplement: Supplementary file 1 [file viruses-15-00283-s001.zip › Supplementary Figure S1.pdf]

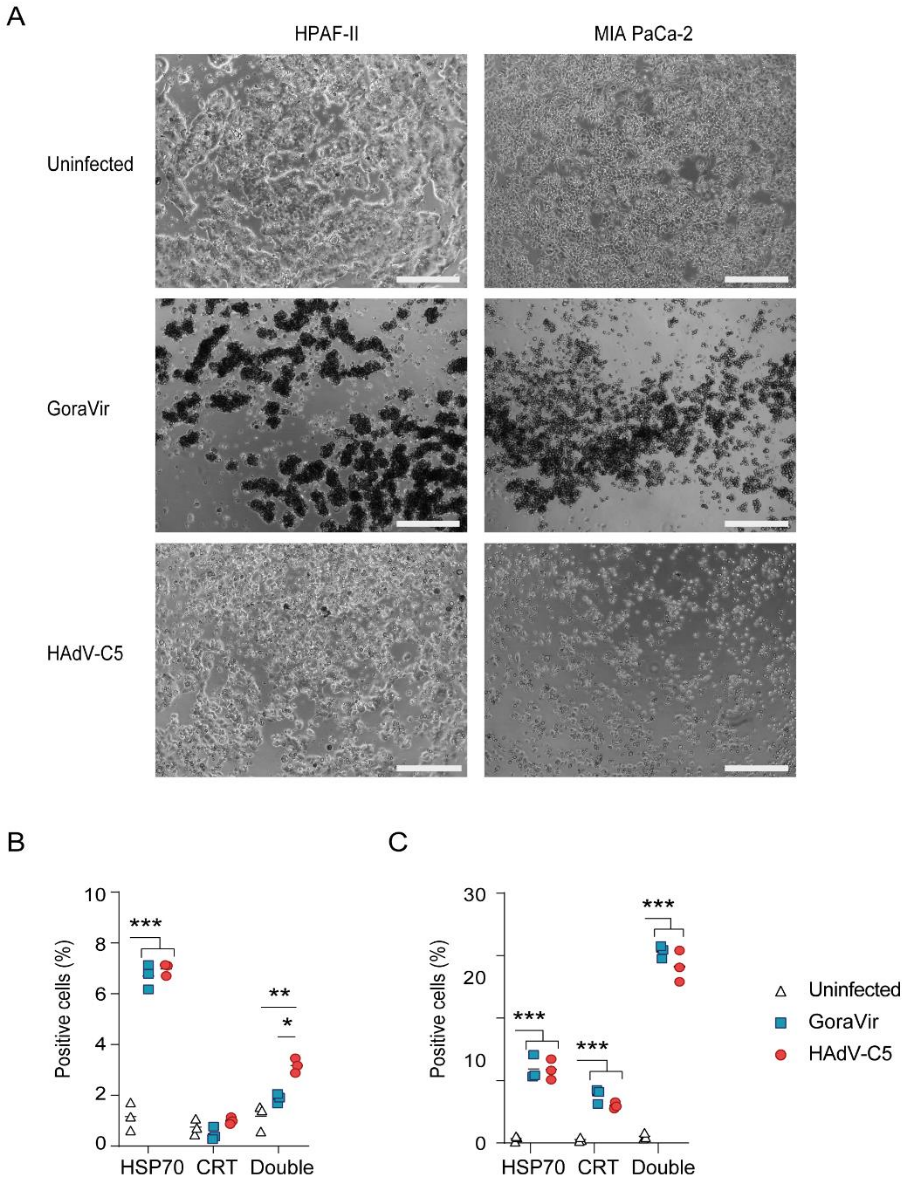


**Supplementary Figure S1. Protein expression of immunogenic cell death (ICD) markers in HPAF-II and MIA PaCa-2 upon infection with GoraVir and HAdV-C5.** A) Light microscopy images of HPAF-II and MIA PaCa-2 cells infected with GoraVir or HAdV-C5 at MOI 10 and 48 hpi. Scale bar represents 500µm; B) Percentage of CRT-positive, HSP70-positive, or double-positive HPAF-II and C) MIA PaCa-2 cells as determined by flow cytometry. Mean is depicted of n=3 biologically independent replicates. Means were compared one-way ANOVA with Tukey correction for multiple testing. Significant differences are indicated by asterisks, with p values < 0.05 shown as *, <0.01 shown as **, and <0.001 shown as ***.
