# Supplementary material for: Immunostimulatory Profile of Cancer Cell Death by the AdV-Lumc007-Derived Oncolytic Virus ‘GoraVir’ in Cultured Pancreatic Cancer Cells
Source: Viruses. 2023 Jan 19;15(2):283. doi: 10.3390/v15020283 (PMC9959036; doi:10.3390/v15020283)
Supplement: Supplementary file 1 [file viruses-15-00283-s001.zip › Supplementary Figure S2.pdf]

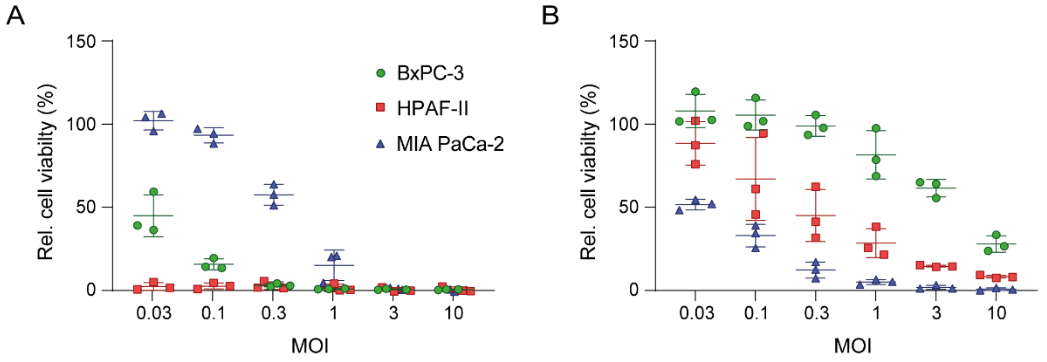

Supplementary Figure S1. Supplementary Figure S2. Cell viability of PDAC cell lines upon infection with GoraVir and HAdV-C5. BxPC-3, HPAF-II, and MIA PaCa-2 cells were infected with A) GoraVir or B) HAdV-C5 at various MOI and cell viability was measured at 6 days post infection. Represented is the mean cell viability relative to uninfected cells. Depicted are mean and SD of n=3 biologically independent experiments each performed in triplicate.
